# Regulating Diet

**Published:** 1883-07

**Authors:** 


᾿# HALL'S JOURNAL OF HEALTH

AND

MISCELLANY.

MONTHLY.

E. H. GIBBS, A. M., M. D., EDITOR.

FOUNDED IN 1854.

### REGULATING DIET.

WHEN a piece of land is exhausted of the element which is the principal ingredient of a certain crop, that ingredient must be supplied, or the crop will fail in quantity and quality; hence the thrifty farmer ascertains the wants of the soil, and supplies it with the needed fertilizer each year.

The human body is exhausted of its elements day by day, and day by day must these elements be supplied by what we eat and drink. But the required proportion of these elements changes with the seasons, with the temperature of the weather; and he who eats the same in quantity or quality in July as at Christmas, does himself an injury. All food contains two chief principles: Carbon, to keep from freezing; Nitrogen, to keep from famishing. The proportion of these elements varies with the food. Those who work a great deal require a great deal of nourishment, of nitrogen, for it is the flesh-forming principle. Those who are exposed a great deal to the cold should eat the carbonaceous, the heat-supplying foods. Butter and fat are three-fourths carbon; vegetables have but little, berries none. Hence Greenlanders, in their icy homes, luxuriate in blubber and whale oil, while the people of the South revel in oranges and bananas, on the plantain and the peach, on dates and figs, on lemons, tamarinds, pine-apples, on alligator pears, bread fruit, and the luscious mango and cherimoyer.

We who live in latitudes between, are permitted the diet of the Polar Sea and the tropics in their season.

A wise man will take but little carbonaceous food on a suddenly hot day; but if suddenly cold, it is best for him to eat more of fuel-making food. An infinite number of fevers and of colds would be avoided if timely attention were paid to these things. By the aid of these statements the following tables may be used to great advantage, showing the amount of carbon or of heat-producing principle in several articles of food. There is not one per cent. of nitrogen, of flesh-forming principle in fruits, berries, and the more common vegetables. Meats have about fifteen per cent. The meats average twenty-five per cent. of nutriment, that is, including both carbon and nitrogen. Of all meats mutton is the most nutritious—thirty per cent. ; fish least, twenty per cent. Of all vegetables, white beans are the most nutritious, ninety-five per cent. ; wheat flour, ninety per cent. ; turnips, the least, five per cent. Of fruits, plums are the most nutritious, thirty per cent.; apples, seventeen ; melons and cucumbers, three ; the rest being mere water and waste. The more waste the more open the bowels are.

THE FOLLOWING TABLE SHOWS THE PERCENTAGE OF CARBON :

| | | | | | |
|---|---|---|---|---|---|
| Apricots | 0 | Potatoes | 11 | Wheat Bread | 40 |
| Berries | 0 | Lean Meat | 13 | Sugar | 42 |
| Cherries | 0 | Rye Bread | 31 | Apples | 45 |
| Currants | 0 | Gum Arabic | 36 | Fat meats | 53 |
| Turnips | 3 | Arrow Root | 36 | Butter | 65 |
| Artichokes | 9 | Green Peas | 36 | Soup | 75 |
| Blood | 10 | Starch | 37 | Lard | 80 |
| Milk | 10 | Lentils | 37 | Beans | 88 |

